# Water constraints drive allometric patterns in the body shape of tree frogs

**DOI:** 10.1038/s41598-020-80456-1

**Published:** 2021-01-13

**Authors:** Kathleen M. S. A. Castro, Talita F. Amado, Miguel Á. Olalla-Tárraga, Sidney F. Gouveia, Carlos A. Navas, Pablo A. Martinez

**Affiliations:** 1 Programa de Pós-Graduação em Ecologia e Conservação, Universidade Federal de Sergipe, São Cristóvão, Sergipe 49.000-100, Brazil; 2 Departamento de Biología, Geología, Física y Química Inorgánica, Universidad Rey Juan Carlos, Tulipán s/n, Móstoles, 28933 Madrid, Spain; 3 Departamento de Fisiologia Geral, Instituto de Biociência, Universidade de São Paulo, São Paulo, Brazil; 4 Departamento de Ecologia, Universidade Federal de Sergipe, São Cristóvão, Sergipe Brazil; 5 Departamento de Biologia, Universidade Federal de Sergipe, São Cristóvão, Sergipe Brazil; 6 PIBi Lab – Laboratório de Pesquisas Integrativas em Biodiversidade, Universidade Federal de Sergipe, São Cristóvão, Sergipe 49100-000 Brazil

**Keywords:** Ecology, Evolution

## Abstract

The origin of morphological diversity is a critical question in evolutionary biology. Interactions between the environment and developmental processes have determining roles in morphological diversity, creating patterns through space and over time. Also, the shape of organisms tends to vary with increasing size as a result of those developmental processes, known as allometry. Several studies have demonstrated that the body sizes of anurans are associated with hydric conditions in their environments and that localities with high water stress tend to select for larger individuals. However, how environmental conditions alter those patterns of covariance between size and shape is still elusive. We used 3D geometric morphometric analyses, associated with phylogenetic comparative methods, to determine if the morphological variations and allometric patterns found in Arboranae (Anura) is linked to water conservation mechanisms. We found effects of the hydric stress on the shape of Arboranae species, favouring globular shapes. Also, the allometric patterns varied in intensity according to the water stress gradient, being particularly relevant for smaller frogs, and more intense in environments with higher water deficits. Our study provides empirical evidence that more spherical body shapes, especially among smaller species, reflect an important adaptation of anurans to water conservation in water-constrained environments.

## Introduction

Environmental conditions, both past and present, together with ontogenetic development and evolutionary history are key factors to understand organismal phenotypic variation across space and time^1–4^. Different environments and physiological requirements (e.g., thermoregulation and/or hydroregulation), associated with ecological interactions^5,6^ and micro-habitat utilisation^7^, allow natural selection to act differentially on animal phenotypes^8–10^. Environmental variation is known to drive alterations in developmental processes^11–13^. For example, anurans that breed in ephemeral ponds develop faster than those that breed in permanent ponds and this shorter development time results in smaller adults^14^. As developmental signalling pathways tend to be phylogenetically conserved^15^, it is expected that closely related taxa respond similarly to the same environmental cues. When developmental changes are adaptive, one can expect to observe varying patterns of body size and shape covariation in different environments^16^.

The size and shape of organisms affect nearly all aspects of their biology (e.g., reproduction, biomechanics, and physiology)^17–19^. From a physiological point of view, several studies have focused on understanding the relationships between body size and environmental gradients at different spatial scales^8,20–24^. Carl Bergmann^25^ described an association between increasing body size of endothermic animals towards colder regions and proposed a heat conservation mechanism as an explanation. Bergmann argued that larger bodies act as a selective advantage in cold environments because of their reduced surface area to volume ratios (SA:V). Subsequent studies found that an analogous water-conservation mechanism that relies on varying SA:V ratios can be involved as a selective pressure on the geographic variation of anuran body sizes as well^8–10,23,26,27^. Larger anurans have smaller SA:V ratios, which reduces the surface area available (relative to volume) for evapotranspiration and becomes an advantage to thrive in arid environments. As such, natural selection would favour increasing body sizes of anuran inhabiting regions with high water deficit^8,10,23,27^.

Similar to body size, the shape of an organism can also affect the rate of water loss through evapotranspiration^4,28^. For instance, it has been observed that the more spherical shape of species from the Myobatrachidae family reflects adaptations to dry conditions^4^. The rounded shape of these animals can be explained, at least in part, by the fact that more globular organisms have smaller surface areas exposed to the environment, thus reducing water loss^4^. Within this perspective, few studies have considered the importance of shape for the osmotic and thermal equilibria and those have mostly used SA:V ratios as a proxy for shape^8,20^. Other measurements of shape remain largely unexplored. Body shape also influences the distribution of organs involved in the pumping and distribution of blood (e.g., heart and vascular lung)^29^, and interestingly, blood distribution is an important factor in regulating the temperature of ectothermic animals^30^. Additionally, the shape is associated with the rate at which processes such as the diffusion of matter and thermal conduction occurs^17^. In this case, the area of evaporative surface (e.g., the area exposed to the external environment) will be inversely related to the distance that the material or energy must traverse across the body^17,31,32^. Then, animals with the same SA:V, but different shapes (e.g., globular or elongated), would respond differently to water loss, as long as this attribute affects the rate of water flow and the heating rate of the body. As such, more rounded bodies would confer an advantage in terms of water economy by decreasing both water loss and heating rates.

Some adaptive changes in body proportions require changes in size for an organism to survive^33^. The covariance of shape and size, as a result of developmental processes in organisms, is known as allometry^33–35^. Allometry is widespread in nature and can be observed throughout the ontogeny of an individual, between individuals in a population at the same ontogenetic stage, and between different species^34,35^. Given that environmental conditions over evolutionary timescales can drive size and shape, it would be expected that such ecological processes could also affect allometric patterns^1,36,37^. Changes in allometric patterns, for example, can occur if selective forces act differently on traits or body sizes that occur in different environmental conditions^1^. If so, the role of the environment on shifting the size-shape correlation may define which crucial relationship between these two traits is maintained through time^37^.

**Figure 1:**
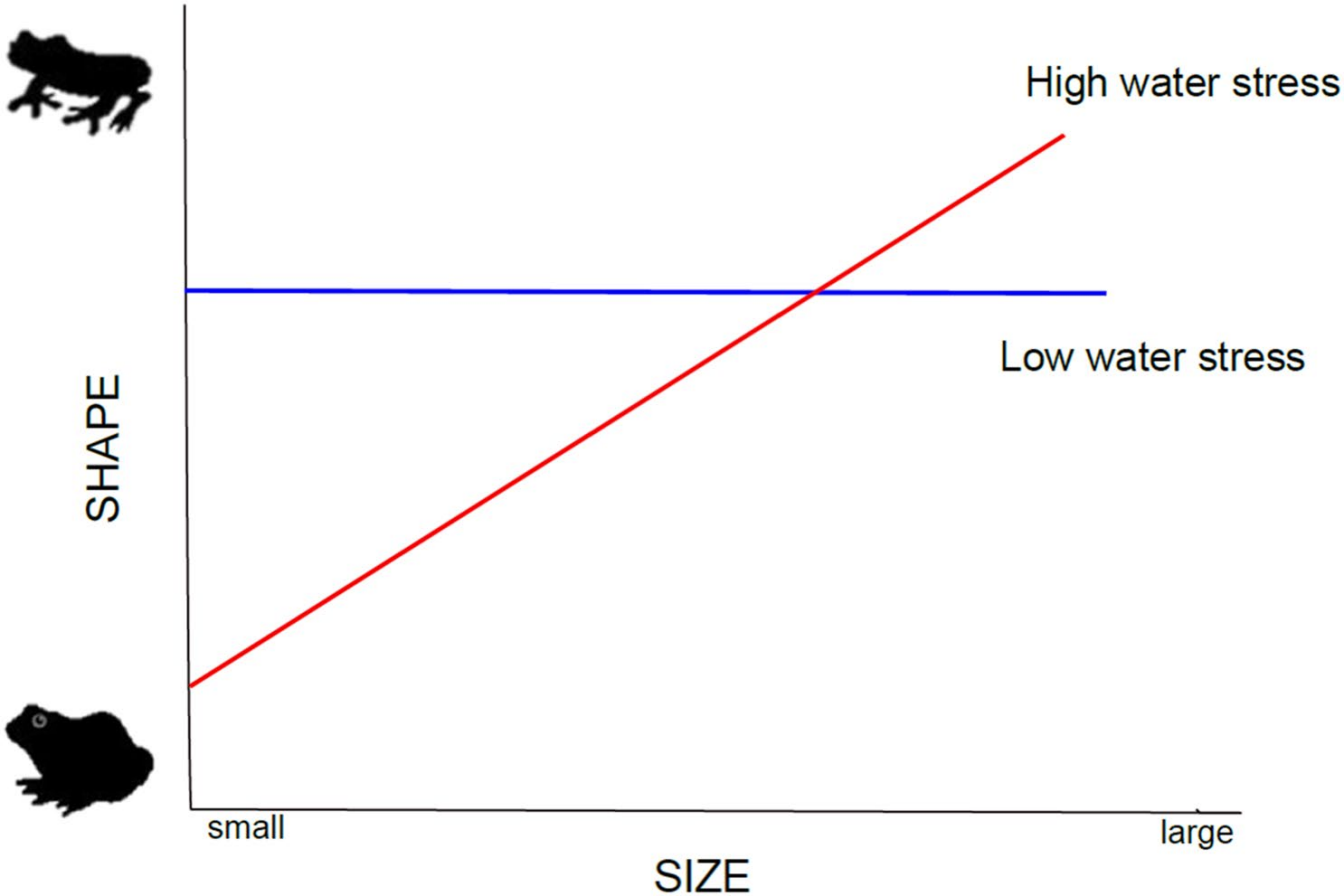
Differential allometry hypothesis. The vertical axis represents body shape and the horizontal axis body size. The red line represents the expected allometric pattern for environments with high water stress since smaller species are expected to have a more globular body shape than larger species. Alternatively, the blue line represents the expected isometric or slight allometric pattern for environments with low water stress.

The Arboranae clade is the most diversified group of anurans and is currently divided into three major families: Hylidae; Phyllomedusidae, and Pelodryadidae^38^. Arboranae comprises approximately 45 genera and more than 800 species distributed in the Americas, Eurasia, Australia, and Papua New Guinea^28^. Their broad taxonomic and environmental diversity makes them an ideal group for analysing the effects of water availability on the covariance of shape and size. We evaluated whether water availability has been a factor in the evolution of body shape in Arboranae species. Specifically, we explored whether species exposed to environments with low water vapor pressures tend to have a more spherical shape (the *shape hypothesis*), and analysed if their allometric patterns in shape become stronger in arid environments where small species show more spherical body shapes as compared to the larger ones (the *differential allometry hypothesis*) (Fig. 1).

## Results

**Figure 2:**
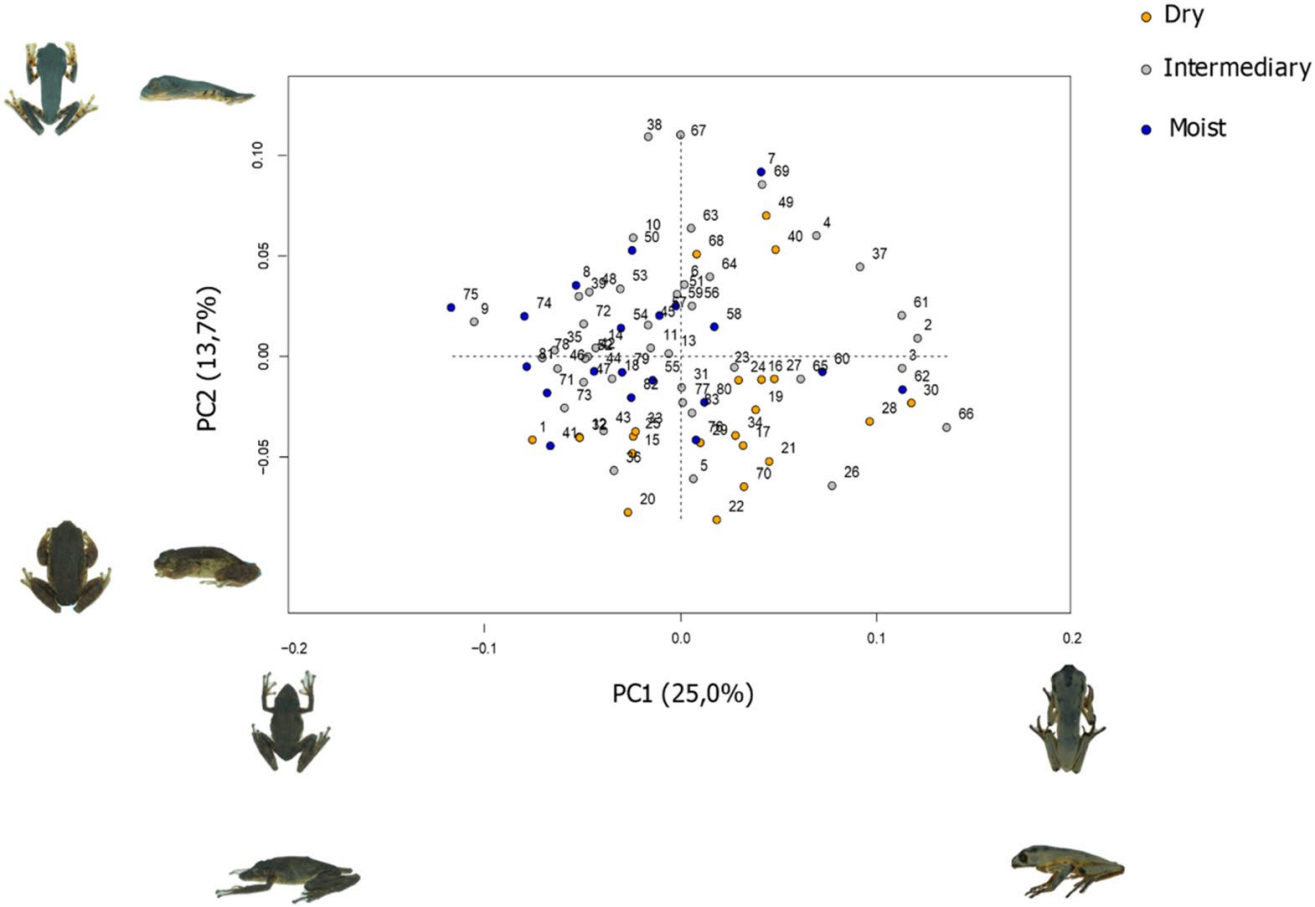
Principal component analysis for the morphospace in Arboranae species. The two axes explain 38.7% of the variation in shape. The horizontal axis is PC1 and the vertical axis is PC2. Colours represent the variation in environmental water availability according to three categories (dry, intermediate, and moist, see text for details). Species on the positive side of PC1 have a shorter snout, whereas species on the negative side display longer snout. Species on the negative side of PC2 have rounder body than species on the positive side. The morphology for each component is to represent by 3D models of Arboranae (PC1 positive: *Phyllomedusa distincta,* SVL = 50.66 mm; PC1 negative: *Scinax garbei*, SVL = 48.40; PC2 positive: *Callimedusa tomopterna,* SVL = 43.14; PC2 negative: *Bokermannohyla pseudopseudis*, SVL = 52.5).

The body sizes and shapes of the species exhibited low, but significant, phylogenetic structuring (K-mult = 0.36 and K-mult = 0.28, respectively; *p* < 0.01). The first two principal components of shape (PC1 and PC2) explained 38.7% of the observed morphological variation between species, with PC1 being more related to head shape (Fig. 2). Species that occupied the negative side of PC1 in the morphospace demonstrated a more tapered rostrum as compared to species on the positive side of this axis (Fig. 2). PC2 was more associated with body shape, with species showing negative values for PC2 having more globular bodies than those on the positive side of the axis, which had more elongated shapes (Fig. 2). Species occupying high water stress regions displayed predominantly negative PC2 values in the morphospace (i.e. more globular bodies).

**Figure 3:**
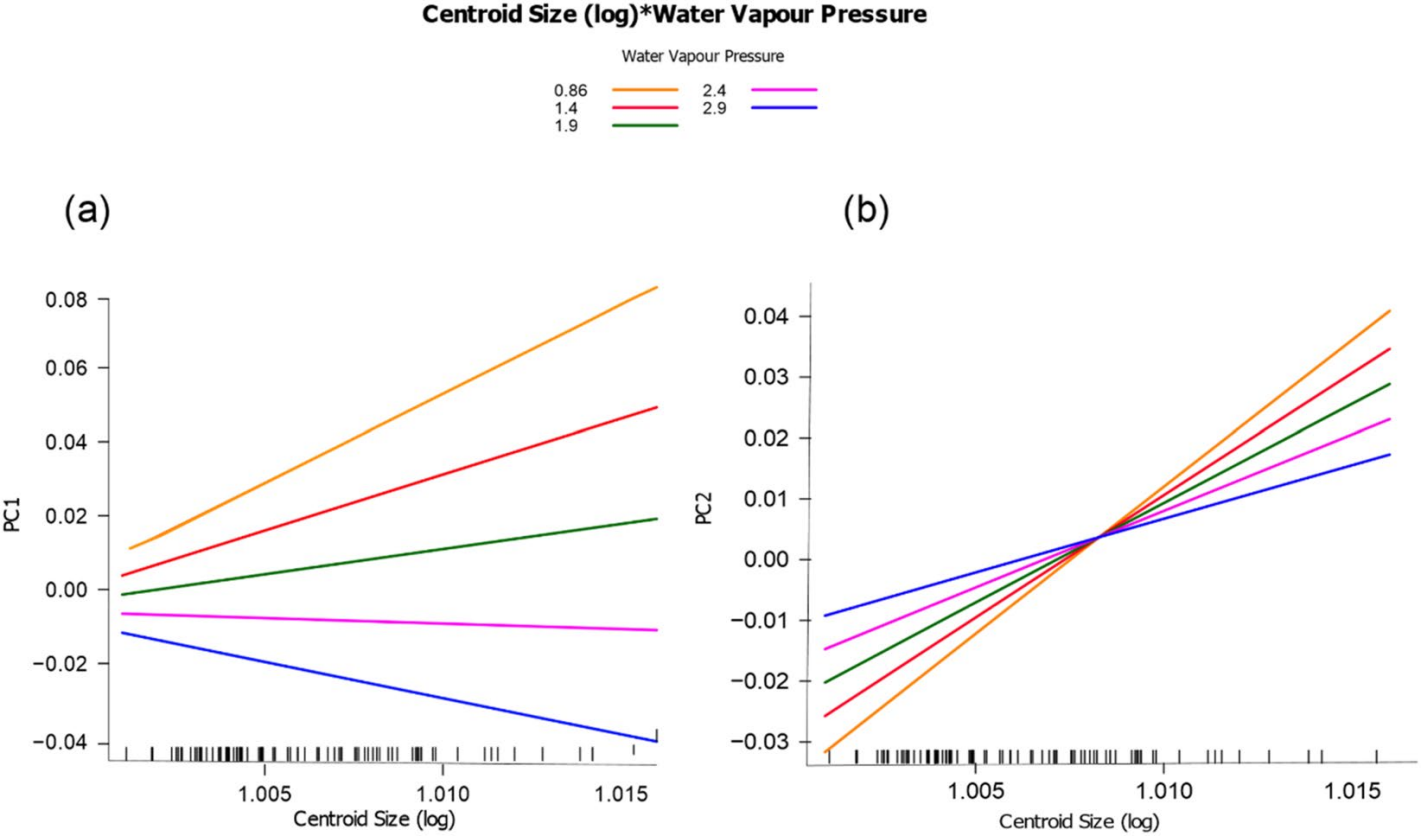
Phylogenetic regression between log-transformed centroid size (Cs) and principal components (PC1 and PC2) of the shape of Arboranae species across a water stress gradient. (**a**) Relationship between Cs and PC1; (**b**) relationship between Cs and PC2. Colour scale varies from dry (orange) to more humid (blue) environments. The bars on the x-axis are sampled data points.

Our results indicated an evident allometric pattern in the Arboranae group (*p* < 0.05) (Table 1). We also observed that the environment affects the morphology of the species (*p* < 0.05), as evidenced by their distributions within the morphospace (Fig. 2). Additionally, we found significant differences when we compared allometric patterns across different water deficit levels (*p* < 0.05) (Table 1; Fig. 3). The observed variance of the first two PCs of shape in relation to body size indicated the existence of variations in the intensities of the covariance of shape and size because of water gradients. Both PCs (PC1 and PC2) showed increases in the intensities of allometric patterns with increasing water deficit conditions. As such, in environments with higher water constraints, smaller species tended to show more globular shapes (negative PC2 values) than larger species (positive PC2 values).

**Table 1:** Phylogenetic MANCOVA regression for procrustes shape variables.

Predictor	Df	SS	F	Z	*p* value
Log_10_(Cs)	1	0.001559	2.8942	2.2846	0.024
WVP	1	0.001298	2.4104	2.0665	0.026
Cs:WVP	1	0.001885	3.4999	2.7198	0.007
Residual	79	0.042558			
Total	82	0.047301			

*Cs* centroid size; *WVP* water vapour pressure; *Df* degree of freedom; *SS* sums-of-square; *F* Cohen's F; *Z* scores (effect-size).

The analysis of morphological disparity based on the variance of Procrustes revealed that the shape of species tends to vary in distinct manners according to size (*p* < 0.05). Morphological variation was relatively little within smaller-sized lineages (0.006) as compared to medium-sized (0.011) or large-bodied (0.021) lineages, an evidence that body shape tends to be more constrained in small species.

## Discussion

Our study shows the existence of variation in body shape and allometric patterns among anuran species in the Arboranae clade that occupy different positions across an environmental gradient of water availability. Lineages under higher water stress tend to be more globular and have more discernible allometric patterns of their body shape. Also, smaller species tend to be relatively more globular than large-sized species. Therefore, our results support both the *shape* and *differential allometry* hypotheses*. *Furthermore, we found that both body size and shape display weak phylogenetic signals in this clade, indicating that these traits tend to vary more than expected by the phylogenetic history of Arboranae^39^. In this regard, anurans are known to vary little in body size compared to other vertebrates^40^, suggesting high lability of body dimensions in response to abiotic selective pressures within the morph-physiological limits of the group^41^.

The globular body shape of species, especially of the smaller ones that inhabit arid environments, reflects the importance of water retention processes. Under the water constraints of drier environments, being smaller represents a challenge to avoid water loss, and anurans are selected to enhance some water-conserving strategies. These strategies often include an increase in body size—which allows a slower rate of warming and dehydration via decreasing SA:V ratios—or to increase skin resistance to water loss to prevent desiccation^9^. Also, the more spherical shapes of anurans that inhabit dry habitats as a mechanism to conserve water are supported under biophysical and physiological reasonings. Terrestrial anurans absorb water mainly from specialized cells at the ventral skin, in response to the osmotic gradient of the tissues. This water content is then dragged from the tissue to the exterior surface through a highly permeable skin, thus creating a vapour pressure gradient^42,43^. Consequently, a more globular shape increases the average distance between inner tissues and the external environment, which confers a double benefit. Firstly, the thicker tissue layer slows the diffusion and the loss of water due to the resistance against the water vapour gradient. Secondly, more globular animals will require more time to warm up, slowing the increase of latent water heat and, thus the loss of viscosity of its internal water that further reduces the rate of diffusion. Therefore, a globular shape may be a crucial adaptation for survival in arid environments, especially for smaller species.

The allometric relationship between size and shape in the Arboranae clade (Table 1, Fig. 3) indicates that the morphological variations observed in arboreal anurans is largely explained by variations in their body size. Many morphological variations are, in fact, strongly associated with the effects of allometric scale^33,35^, which has been well reported among different lineages of vertebrates (e.g., bony fish, salamanders, and caecilians)^1,15,44^. Therefore, our results reinforce the importance of allometry as one of the processes that modulate the patterns of phenotypic diversity in living organisms. Moreover, comparisons of the allometric trajectories of Arboranae species showed that allometric patterns for this group were not homogeneous, as they varied in response to water deficit gradients (Fig. 3). This result highlights that natural selection can favour specific allometric relationships, depending on the environmental context of the species, and thus promote divergent trajectories among different lineages. In arid regions, selective pressures on anuran size and shape appear to be mainly driven by the physiological processes related to the water economy, which generate more intense allometric patterns than those detected in regions with low water restrictions. It is worth mentioning that our study does not consider microclimate issues, such as the GPS point at the ground level or the coverage, which could be important as well on the evolution of the allometric pattern of arboreal anurans.

Water is a vital element for all living organisms, and especially for amphibians, in which both physiological (gas exchange, osmoregulation, and thermoregulation) and ecological (reproduction, feeding, and shelter) aspects are immediately affected by its availability^28^. Rates of water loss through evapotranspiration among amphibians are affected by environmental temperatures and the relative humidity of the air, creating vapour pressure gradients between the animals and their environments^9,27,32^. Amphibians living in arid regions are subject to strong water vapour gradients with their environment due to high temperatures and low humidity (low water vapour pressure in the environment), increasing evapotranspiration^28^. A variety of strategies reducing water losses in anuran amphibians inhabiting dry environments have been selected over evolutionary time, including cutaneous wax secretions, selection of humid microhabitats, estivation, aggregation behaviour, and cocoon formation^45^. Species inhabiting humid regions are not, however, under the same water restrictions as species inhabiting arid environments. Elevated temperatures and high humidity (high water vapour pressure in the environment) in humid regions reduce the water vapour gradients between those animals and their environment, significantly reducing their rates of evapotranspiration^28^. Thus, anurans inhabiting humid regions would be expected to experience lower selection pressure for attributes related to water retention than anurans from drier regions.

In summary, our study revealed that the body shape of tree frogs (Arboranae clade) varies in response to the evaporative capacity of the environment in which smaller frogs are more prone to changes in shape than larger ones. This scaling pattern is also a consequence of the environmental gradient in water availability, with allometric relationships being stronger in arid regions. Accordingly, our two hypotheses of *shape* and *differential allometry* were corroborated. The variation in body shape from a slender to a more globular one, particularly in smaller species in drier conditions, provides empirical evidence of an alternative adaptive pathway to environmental constraints linked to water retention among anurans. This study emphasises the importance of examining allometric patterns to understand better the role of size in the expression of adaptive strategies of organisms to the physical constraints of the environment.

## Methods

### Specimen data collection

A total of 195 specimens belonging to 83 species in the Arboranae clade were analysed, including Hylidae (n = 62), Pelodryadidae (n = 5), and Phyllomedusidae (n = 16). The sample size for each species varied between one and nine specimens (mean = 3), as determined by the availability of specimens in the collections (Supplementary Table S1). To avoid problems related to sexual dimorphism, we consider only adult females, which were identified by the absence of male sexual traits (vocal sacs, pre-polex spines, and nuptial pads). Measured specimens were from the London Natural History Museum (United Kingdom), the Museum für Naturkunde (Germany), the Herpetological Collection at the Federal University of Sergipe (Brazil), and the Herpetological Collection of the Amazonian Research Institute (Brazil). In this study, live specimens were not used.

### Reconstruction of the specimens in 3D models

**Figure 4:**
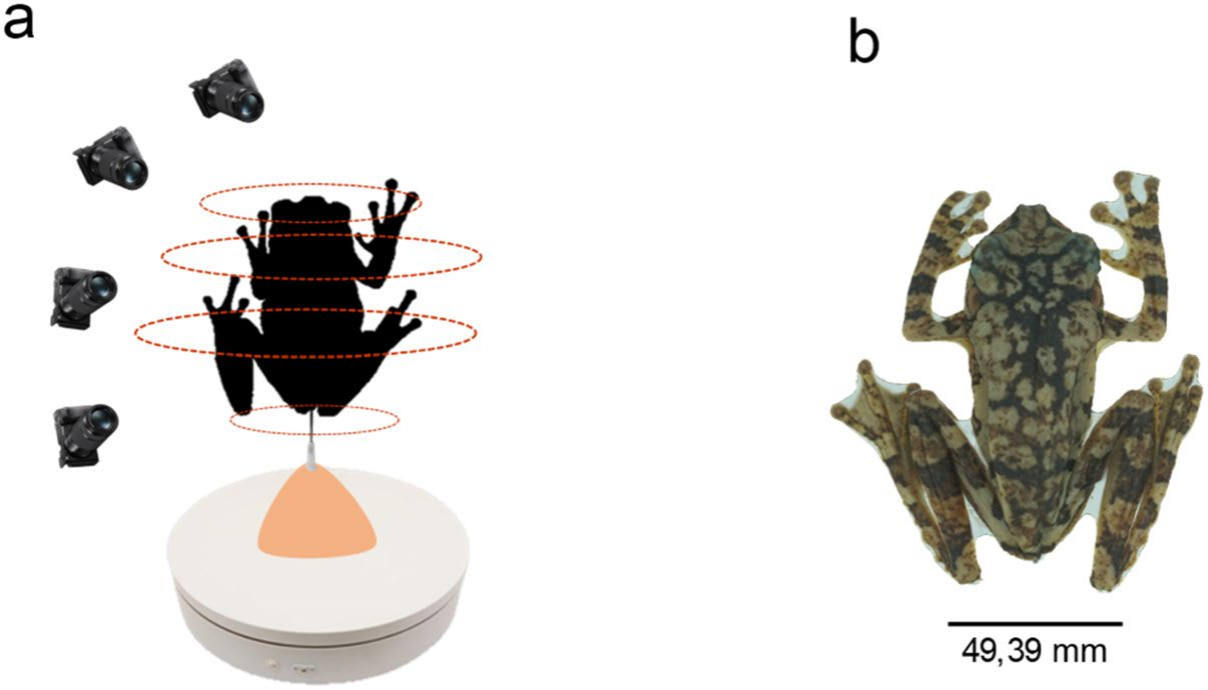
Reconstruction of 3D models using the photogrammetry method. (**a**) The first step is to position the samples to capture the photos from four different angles. (**b**) After the 2D image overlay step by Autodesk Recap Photo software version 20.1.0.32 (https://www.autodesk.com), we finally obtained our 3d models.

We used a photogrammetric technique, following the protocols proposed by Amado et al.^20^ to reconstruct the specimens as 3D models. The method builds three-dimensional models from two-dimensional images (Fig. 4) in three steps: (i) positioning the specimens, (ii) capturing the images, and (iii) constructing the 3D model*.* Each anuran specimen was positioned vertically on a turntable and supported by a pin positioned in the cloaca and fixed on a plastic base (Fig. 4a). Each specimen was then photographed from four different angles to record it in different positions (Fig. 4a) using a Nikon D3400 digital camera (with a 50 mm lens and the configurations ISO 100 and F11). We used Foldio 360 software to aid in capturing the images, connecting it by Bluetooth to the rotating platform. A total of 76 images were captured of each specimen. The reconstructions of the specimens as 3D models were performed using Autodesk Recap Photo software version 20.1.0.32 (https://www.autodesk.com) (Fig. 4b). The snout-to-vent length (SVL) of each individual was measured by a digital calliper to calibrate each model.

### Distribution and environmental data

We collected occurrence records (longitude and latitude) for each species using the Global Biodiversity Information Facility online database (GBIF—http://www.gbif.org) and from localities listed in the Amphibian Web database (https://amphibiaweb.org). All locality data were carefully examined to check for possible referencing errors, and any duplicate or dubious records were removed.

As a descriptor of evapotranspiration capacity, we used the water vapour pressure (WVP), which considers both temperature and water availability in the environment. Lower values of WVP in the environment reflect higher rates of water loss. We then estimated annual mean vapour pressure of water (kPa) for each Arboranae species based on locality records, using WVP as a measure of environmental water stress. WVP data were obtained from the WorldClim—*Global Climate Database* (https://worldclim.org, March 2020) at a 10 arcmin resolution (~ 18 km). The WVP values varied from 0 to 3; the larger values represent environments with high vapour pressure levels. We then used those vapour pressure values to categorise environments into three levels of water stress: high stress = 1st quartile; intermediate stress = 2nd and 3rd quartile; low stress = 4th quartile).

### Morphological data acquisition

**Figure 5:**
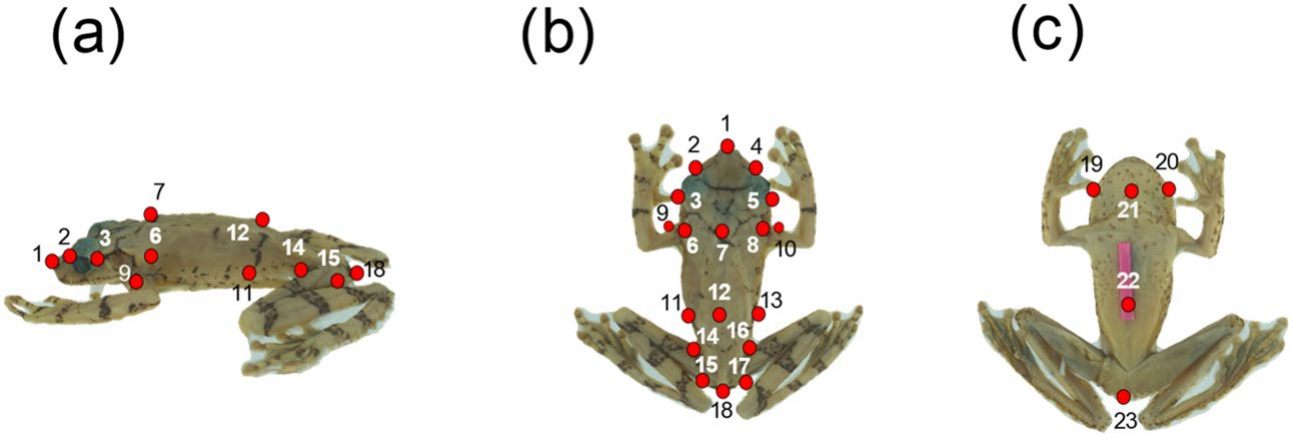
Landmarks used to characterise body shape of species in the Arboranae clade. An overall of 23 landmarks were inserted into the body of each specimen. (**a**) Lateral view; (**b**) dorsal view and (**c**) ventral view.

To evaluate how the shapes of anuran species covaried with size and with WVP of the environment, we utilised 3D geometric morphometric methods. A set of 23 homologous landmarks were digitised on different regions of individual anuran bodies (dorsal and ventral) to capture morphological information that could be associated with water retention processes. Those landmarks were digitised in the head region (n = 11), on upper appendages (n = 2), the pelvic region (n = 4), inferior appendages (n = 4), and the cloaca (n = 2) (Fig. 5). All of the points were chosen to identify bony regions that would demonstrate only low deformation during fixation processes. We used the Landmark Editor v.3.0 program to insert the landmarks^46^. Digitalisation of the landmarks was performed by only a single person to avoid possible distortion errors. The coordinates of the landmarks were obtained using the Procrustes generalised superimposition method^47^, removing the effects of size, position, and orientation. We estimated the morphological mean of each of the species using the morphological coordinates extracted by the Procrustes method. We also obtained data concerning size for all specimens based on the size of the centroid on a logarithmic scale. The centroid size (Cs) was calculated as the square root of the sum of the squared distances of each reference point to the centroid^48^.

### Morphological data analysis

First, we analysed the effects of phylogenetic structuring on the shapes and sizes of species using the K-mult index^49^, which varies from 0 to 1; values near 0 indicate the absence of phylogenetic structure, while values near 1 reflect a strong phylogenetic signal. Subsequently, we performed a principal component analysis (PCA) to explore the shapes of the species in different environments (of low, intermediate, and high-water stress), projecting into the morphospace the first two principal components (PCs) that explained most of the observed morphological variation. To estimate the effects of body size (evolutionary allometry) and water vapour pressure (WVP) on the shape of species, we performed a phylogenetic MANCOVA. We also evaluated the interactions between body size and WVP with body shapes of the species to determine if there were differences in the allometric trajectories in the different environments. In our comparative analyses, we used the phylogeny proposed by Jetz and Pyron^50^.

Finally, we performed an analysis of morphological disparity to establish how shapes vary among different sizes. For this purpose, we divided the species into quartiles according to the Cs (small = 1st quartile, medium = 2nd, 3rd quartile, and large = 4th quartile) and analysed the variance obtained by the Procrustes method for each size category. All morphological analyses were performed using the *geomorph* version 3.2.1 package^51^, the graphic representation of allometric regression was produced using the *effects* version 4.1–4 package^52^ the R platform version. 3.6.1^53^. A significance level α = 0.05 was adopted for all statistical analyses.

## Supplementary Information

Supplementary Information.
