# Supplementary material for: Water constraints drive allometric patterns in the body shape of tree frogs
Source: Sci Rep. 2021 Jan 13;11:1218. doi: 10.1038/s41598-020-80456-1 (PMC7806824; doi:10.1038/s41598-020-80456-1)
Supplement: Supplementary file 1 — Supplementary Information. [file 41598_2020_80456_MOESM1_ESM.docx]

SUPPLEMENTARY INFORMATION

**Water constraints drive allometric patterns in the body shape of tree frogs**

Kathleen M. S. A. Castro^1,6^*, Talita F. Amado^2,6^, Miguel Á. Olalla-Tárraga^2^, Sidney F. Gouveia^4,6^, Carlos A. Navas^3^, Pablo A. Martinez^5,6^

Table S1. List of anurans specimens utilised for analysis. The list is organised from A to Z according to the name of the species.

| Number of specimes | Family | Subfamily | Genus | Species | Collection | Catalog Number | SVL(mm) |
| --- | --- | --- | --- | --- | --- | --- | --- |
| 1 | Hylidae | Acridinae | *Acris* | *Acris crepitans* | NHMUK | 1098 | 25.97 |
| 2 | Hylidae | Acridinae | *Acris* | *Acris crepitans* | NHMUK | 1100 | 24.29 |
| 3 | Phyllomedusidae |  | *Agalychnis* | *Agalychnis dacnicolor* | NHMUK | 1914128236 | 63.06 |
| 4 | Phyllomedusidae |  | *Agalychnis* | *Agalychnis dacnicolor* | NHMUK | 8211154950 | 69.53 |
| 5 | Phyllomedusidae |  | *Agalychnis* | *Agalychnis dacnicolor* | NHMUK | 19329131 | 72.24 |
| 6 | Phyllomedusidae |  | *Agalychnis* | *Agalychnis moreletii* | NHMUK | 19021282830 | 65.02 |
| 7 | Phyllomedusidae |  | *Agalychnis* | *Agalychnis moreletii* | NHMUK | 71112248 | 69.67 |
| 8 | Phyllomedusidae |  | *Agalychnis* | *Agalychnis moreletii* | NHMUK | 7962614 | 65.93 |
| 9 | Hylidae | Lophyohylinae | *Aparasphenodon* | *Aparasphenodon brunoi* | NHMUK | 194041414 | 66.53 |
| 10 | Hylidae | Lophyohylinae | *Aparasphenodon* | *Aparasphenodon brunoi* | NHMUK | 19611660 | 72.38 |
| 11 | Hylidae | Cophomantinae | *Bokermannohyla* | *Bokermannohyla pseudopseudis* | INPA | 1494 | 52.5 |
| 12 | Phyllomedusidae |  | *Callimedusa* | *Callimedusa atelopoides* | INPA | 26586 | 46.58 |
| 13 | Phyllomedusidae |  | *Callimedusa* | *Callimedusa atelopoides* | INPA | 31961 | 37.42 |
| 14 | Phyllomedusidae |  | *Callimedusa* | *Callimedusa tomopterna* | INPA | 6505 | 43.14 |
| 15 | Phyllomedusidae |  | *Callimedusa* | *Callimedusa tomopterna* | INPA | 6508 | 41.40 |
| 16 | Phyllomedusidae |  | *Cruziohyla* | *Cruziohyla craspedopus* | INPA | 3747 | 74.75 |
| 17 | Phyllomedusidae |  | *Cruziohyla* | *Cruziohyla craspedopus* | INPA | 3746 | 61.57 |
| 18 | Hylidae | Dendropsophinae | *Dendropsophus* | *Dendropsophus elegans* | UFS | C652 | 28.47 |
| 19 | Hylidae | Dendropsophinae | *Dendropsophus* | *Dendropsophus leucophyllatus* | INPA | 31379 | 31.28 |
| 20 | Hylidae | Dendropsophinae | *Dendropsophus* | *Dendropsophus leucophyllatus* | INPA | 31378 | 30.22 |
| 21 | Hylidae | Dendropsophinae | *Dendropsophus* | *Dendropsophus leucophyllatus* | INPA | 31366 | 29.50 |
| 22 | Hylidae | Dendropsophinae | *Dendropsophus* | *Dendropsophus marmoratus* | INPA | 3759 | 33.68 |
| 23 | Hylidae | Dendropsophinae | *Dendropsophus* | *Dendropsophus marmoratus* | INPA | 3760 | 39.64 |
| 24 | Hylidae | Dendropsophinae | *Dendropsophus* | *Dendropsophus parviceps* | INPA | 33755 | 27.23 |
| 25 | Hylidae | Dendropsophinae | *Dendropsophus* | *Dendropsophus parviceps* | INPA | 26598 | 25.46 |
| 25 | Hylidae | Dendropsophinae | *Dendropsophus* | *Dendropsophus parviceps* | INPA | 20443 | 28.72 |
| 26 | Hylidae | Dendropsophinae | *Dendropsophus* | *Dendropsophus parviceps* | INPA | 28662 | 19.12 |
| 27 | Hylidae | Dendropsophinae | *Dendropsophus* | *Dendropsophus parviceps* | INPA | 28665 | 20.8 |
| 28 | Hylidae | Dendropsophinae | *Dendropsophus* | *Dendropsophus sarayacuensis* | INPA | 28680 | 31.86 |
| 29 | Hylidae | Hylinae | *Dryophytes* | *Dryophytes arenicolor* | NHMUK | 18922874 | 37.13 |
| 30 | Hylidae | Hylinae | *Dryophytes* | *Dryophytes arenicolor* | NHMUK | 190661117 | 40.82 |
| 31 | Hylidae | Hylinae | *Dryophytes* | *Dryophytes arenicolor* | NHMUK | 191112424 | 33.12 |
| 32 | Hylidae | Hylinae | *Dryophytes* | *Dryophytes arenicolor* | NHMUK | 190112198385 | 41.16 |
| 33 | Hylidae | Hylinae | *Dryophytes* | *Dryophytes arenicolor* | NHMUK | 1914128229230 | 41.08 |
| 34 | Hylidae | Hylinae | *Dryophytes* | *Dryophytes avivocus* | NHMUK | 19614451 | 34.75 |
| 35 | Hylidae | Hylinae | *Dryophytes* | *Dryophytes avivocus* | NHMUK | 19614452 | 34.35 |
| 36 | Hylidae | Hylinae | *Dryophytes* | *Dryophytes avivocus* | NHMUK | 192511430 | 37.45 |
| 37 | Hylidae | Hylinae | *Dryophytes* | *Dryophyts chrysoscelis* | NHM_Berlin | 48902 | 38.71 |
| 38 | Hylidae | Hylinae | *Dryophytes* | *Dryophyts chrysoscelis* | NHM_Berlin | 489021 | 38.54 |
| 39 | Hylidae | Hylinae | *Dryophytes* | *Dryophytes cinereus* | NHMUK | 19571474801 | 46.15 |
| 40 | Hylidae | Hylinae | *Dryophytes* | *Dryophytes cinereus* | NHMUK | 19571474802 | 44.96 |
| 41 | Hylidae | Hylinae | *Dryophytes* | *Dryophytes cinereus* | NHMUK | 184511931 | 48.35 |
| 42 | Hylidae | Hylinae | *Dryophytes* | *Dryophytes eximius* | NHMUK | 188341657 | 20.72 |
| 43 | Hylidae | Hylinae | *Dryophytes* | *Dryophytes eximius* | NHMUK | 195514868892 | 33.18 |
| 44 | Hylidae | Hylinae | *Dryophytes* | *Dryophytes gratiosus* | NHMUK | 19571457591 | 55.93 |
| 45 | Hylidae | Hylinae | *Dryophytes* | *Dryophytes gratiosus* | NHMUK | 1957145792 | 56.64 |
| 46 | Hylidae | Hylinae | *Dryophytes* | *Dryophytes gratiosus* | NHMUK | 19571457593 | 55.17 |
| 47 | Hylidae | Hylinae | *Dryophytes* | *Dryophytes gratiosus* | NHMUK | 1927112317181 | 53.24 |
| 48 | Hylidae | Hylinae | *Dryophytes* | *Dryophytes gratiosus* | NHMUK | 1927112317182 | 51.51 |
| 49 | Hylidae | Hylinae | *Dryophytes* | *Dryophytes japonicus* | NHM_Berlin | 71052 | 36.75 |
| 50 | Hylidae | Hylinae | *Dryophytes* | *Dryophytes japonicus* | NHM_Berlin | 71053 | 33.49 |
| 51 | Hylidae | Hylinae | *Dryophytes* | *Dryophytes plicatus* | NHM_Berlin | 35213 | 44.09 |
| 52 | Hylidae | Hylinae | *Dryophytes* | *Dryophytes squirellus* | NHMUK | 19571460711 | 36.72 |
| 53 | Hylidae | Hylinae | *Dryophytes* | *Dryophytes squirellus* | NHMUK | 19571460712 | 35.04 |
| 54 | Hylidae | Hylinae | *Dryophytes* | *Dryophytes versicolor* | NHM_Berlin | 12082 | 45.26 |
| 55 | Hylidae | Hylinae | *Dryophytes* | *Dryophytes versicolor* | NHM_Berlin | 12083 | 41.59 |
| 56 | Pelodryadidae | Pelodryadinae | *Dryopsophus* | *Dryopsophus aureus* | NHMUK | 589131 | 57.35 |
| 57 | Pelodryadidae | Pelodryadinae | *Dryopsophus* | *Dryopsophus aureus* | NHMUK | 1864102721 | 77.12 |
| 58 | Pelodryadidae | Pelodryadinae | *Dryopsophus* | *Dryopsophus caeruleus* | NHMUK | 18859249 | 98.19 |
| 59 | Pelodryadidae | Pelodryadinae | *Dryopsophus* | *Dryopsophus caeruleus* | NHMUK | 189262112 | 80.16 |
| 60 | Pelodryadidae | Pelodryadinae | *Dryopsophus* | *Dryopsophus caeruleus* | NHMUK | 8491327 | 81.20 |
| 61 | Pelodryadidae | Pelodryadinae | *Dryopsophus* | *Dryopsophus citropus* | NHMUK | 186361680821 | 50.67 |
| 62 | Pelodryadidae | Pelodryadinae | *Dryopsophus* | *Dryopsophus citropus* | NHMUK | 186361680822 | 50.37 |
| 63 | Pelodryadidae | Pelodryadinae | *Dryopsophus* | *Dryopsophus cyclorhynchus* | NHMUK | 19317173791 | 62.65 |
| 64 | Pelodryadidae | Pelodryadinae | *Dryopsophus* | *Dryopsophus cyclorhynchus* | NHMUK | 19317173792 | 67.49 |
| 65 | Pelodryadidae | Pelodryadinae | *Dryopsophus* | *Dryopsophus cyclorhynchus* | NHMUK | 19317173793 | 60.56 |
| 66 | Pelodryadidae | Pelodryadinae | *Dryopsophus* | *Dryopsophus lesueurii* | NHMUK | 1864102722 | 59.28 |
| 67 | Pelodryadidae | Pelodryadinae | *Dryopsophus* | *Dryopsophus lesueurii* | NHMUK | 88731920 | 45.99 |
| 68 | Hylidae | Hylinae | *Hyla* | *Hyla annectans* | NHMUK | 189310938411 | 31.39 |
| 69 | Hylidae | Hylinae | *Hyla* | *Hyla annectans* | NHMUK | 189310938412 | 29.9 |
| 70 | Hylidae | Hylinae | *Hyla* | *Hyla annectans* | NHMUK | 18724171741833 | 37.01 |
| 71 | Hylidae | Hylinae | *Hyla* | *Hyla chinensis* | NHMUK | 18843117 | 34.56 |
| 72 | Hylidae | Hylinae | *Hyla* | *Hyla chinensis* | NHMUK | 189983110 | 34.85 |
| 73 | Hylidae | Hylinae | *Hyla* | *Hyla chinensis* | NHMUK | 19011196 | 34.75 |
| 74 | Hylidae | Hylinae | *Hyla* | *Hyla intermedia* | NHM_Berlin | 59791 | 22.47 |
| 75 | Hylidae | Hylinae | *Hyla* | *Hyla meridionalis* | NHMUK | 184452527 | 40.5 |
| 76 | Hylidae | Hylinae | *Hyla* | *Hyla meridionalis* | NHMUK | 18866293537 | 35 |
| 77 | Hylidae | Hylinae | *Hyla* | *Hyla meridionalis* | NHMUK | 18924183940 | 44.57 |
| 78 | Hylidae | Hylinae | *Hyla* | *Hyla meridionalis* | NHMUK | 188612181620 | 40.56 |
| 79 | Hylidae | Hylinae | *Hyla* | *Hyla meridionalis* | NHMUK | 189154168169 | 46.25 |
| 80 | Hylidae | Hylinae | *Hyla* | *Hyla meridionalis* | NHMUK | 192012038061 | 42.3 |
| 81 | Hylidae | Hylinae | *Hyla* | *Hyla meridionalis* | NHMUK | 192012038062 | 46.05 |
| 82 | Hylidae | Hylinae | *Hyla* | *Hyla meridionalis* | NHMUK | 18841120114116 | 37.52 |
| 83 | Hylidae | Hylinae | *Hyla* | *Hyla meridionalis* | NHMUK | 1928122016917111 | 40.6 |
| 84 | Hylidae | Hylinae | *Hyla* | *Hyla savignyi* | NHMUK | 19388119 | 42.5 |
| 85 | Hylidae | Hylinae | *Hyla* | *Hyla savignyi* | NHMUK | 192781278 | 46.49 |
| 86 | Hylidae | Hylinae | *Hyla* | *Hyla savignyi* | NHMUK | 18648239596 | 35.01 |
| 87 | Hylidae | Hylinae | *Hyla* | *Hyla savignyi* | NHMUK | 193211391140 | 43.35 |
| 88 | Hylidae | Hylinae | *Hyla* | *Hyla savignyi* | NHMUK | 1850102125311 | 37.53 |
| 89 | Hylidae | Hylinae | *Hyla* | *Hyla savignyi* | NHMUK | 1850102125312i | 34.25 |
| 90 | Hylidae | Cophomantinae | *Hypsiboas* | *Hypsiboas albormaginatus* | UFS | C1371 | 51.3 |
| 91 | Hylidae | Cophomantinae | *Hypsiboas* | *Hypsiboas albormaginatus* | UFS | C3715 | 49.6 |
| 92 | Hylidae | Cophomantinae | *Hypsiboas* | *Hypsiboas albormaginatus* | UFS | C3719 | 49.7 |
| 93 | Hylidae | Cophomantinae | *Hypsiboas* | *Hypsiboas albormaginatus* | UFS | C4569 | 55.4 |
| 94 | Hylidae | Cophomantinae | *Hypsiboas* | *Hypsiboas albormaginatus* | UFS | C4664 | 54.3 |
| 95 | Hylidae | Cophomantinae | *Hypsiboas* | *Hypsiboas albopunctatus* | UFS | C3133 | 54 |
| 96 | Hylidae | Cophomantinae | *Hypsiboas* | *Hypsiboas bischoffi* | NHMUK | 889211617 | 55.05 |
| 97 | Hylidae | Cophomantinae | *Hypsiboas* | *Hypsiboas boans* | NHMUK | 72101612131 | 102.38 |
| 98 | Hylidae | Cophomantinae | *Hypsiboas* | *Hypsiboas boans* | NHMUK | 72101612132 | 105.14 |
| 99 | Hylidae | Cophomantinae | *Hypsiboas* | *Hypsiboas calcaratus* | INPA | 24639 | 51.78 |
| 100 | Hylidae | Cophomantinae | *Hypsiboas* | *Hypsiboas calcaratus* | INPA | 26571 | 55.75 |
| 101 | Hylidae | Cophomantinae | *Hypsiboas* | *Hypsiboas callipleura* | NHMUK | 19075733 | 47.86 |
| 102 | Hylidae | Cophomantinae | *Hypsiboas* | *Hypsiboas callipleura* | NHMUK | 191211125 | 51.27 |
| 103 | Hylidae | Cophomantinae | *Hypsiboas* | *Hypsiboas callipleura* | NHMUK | 190368301617 | 43.29 |
| 104 | Hylidae | Cophomantinae | *Hypsiboas* | *Hypsiboas cinerascens* | INPA | 38313 | 34.92 |
| 105 | Hylidae | Cophomantinae | *Hypsiboas* | *Hypsiboas cinerascens* | INPA | 38312 | 36.78 |
| 106 | Hylidae | Cophomantinae | *Hypsiboas* | *Hypsiboas crepitans* | UFS | C1549 | 67.7 |
| 107 | Hylidae | Cophomantinae | *Hypsiboas* | *Hypsiboas crepitans* | UFS | C1551 | 62.2 |
| 108 | Hylidae | Cophomantinae | *Hypsiboas* | *Hypsiboas crepitans* | UFS | C1566 | 63.2 |
| 109 | Hylidae | Cophomantinae | *Hypsiboas* | *Hypsiboas faber* | UFS | C3626 | 101.1 |
| 110 | Hylidae | Cophomantinae | *Hypsiboas* | *Hypsiboas faber* | UFS | C1425 | 98.3 |
| 111 | Hylidae | Cophomantinae | *Hypsiboas* | *Hypsiboas fasciatus* | INPA | 30114 | 51.34 |
| 112 | Hylidae | Cophomantinae | *Hypsiboas* | *Hypsiboas fasciatus* | INPA | 30115 | 49.8 |
| 113 | Hylidae | Cophomantinae | *Hypsiboas* | *Hypsiboas fasciatus* | INPA | 38747 | 44.2 |
| 114 | Hylidae | Cophomantinae | *Hypsiboas* | *Hypsiboas fasciatus* | INPA | 38960 | 45.25 |
| 115 | Hylidae | Cophomantinae | *Hypsiboas* | *Hypsiboas geographicus* | INPA | 25343 | 59.68 |
| 116 | Hylidae | Cophomantinae | *Hypsiboas* | *Hypsiboas lanciformis* | INPA | 38224 | 71.16 |
| 117 | Hylidae | Cophomantinae | *Hypsiboas* | *Hypsiboas lanciformis* | INPA | 38225 | 74.1 |
| 118 | Hylidae | Cophomantinae | *Hypsiboas* | *Hypsiboas lundii* | UFS | C3232 | 56.4 |
| 119 | Hylidae | Cophomantinae | *Hypsiboas* | *Hypsiboas lundii* | UFS | C3248 | 56.5 |
| 120 | Hylidae | Cophomantinae | *Hypsiboas* | *Hypsiboas maculateralis* | INPA | 34687 | 51.74 |
| 121 | Hylidae | Cophomantinae | *Hypsiboas* | *Hypsiboas marginatus* | NHMUK | 1884231315 | 42.15 |
| 122 | Hylidae | Cophomantinae | *Hypsiboas* | *Hypsiboas marginatus* | NHMUK | 19702027 | 45.18 |
| 123 | Hylidae | Cophomantinae | *Hypsiboas* | *Hypsiboas nympha* | INPA | 38318 | 36.74 |
| 124 | Hylidae | Cophomantinae | *Hypsiboas* | *Hypsiboas pombali* | UFS | C4666 | 52.1 |
| 125 | Hylidae | Cophomantinae | *Hypsiboas* | *Hypsiboas raniceps* | UFS | C00522 | 72.1 |
| 126 | Hylidae | Cophomantinae | *Hypsiboas* | *Hypsiboas raniceps* | UFS | C3829 | 77.44 |
| 127 | Hylidae | Lophyohylinae | *Osteocephalus* | *Osteocephalus buckleyi* | INPA | 25618 | 45.13 |
| 128 | Hylidae | Lophyohylinae | *Osteocephalus* | *Osteocephalus buckleyi* | INPA | 25616 | 47.11 |
| 129 | Hylidae | Lophyohylinae | *Osteocephalus* | *Osteocephalus buckleyi* | INPA | 25617 | 49.39 |
| 130 | Hylidae | Lophyohylinae | *Osteocephalus* | *Osteocephalus cabrerai* | INPA | 38343 | 69.00 |
| 131 | Hylidae | Lophyohylinae | *Osteocephalus* | *Osteocephalus cabrerai* | INPA | 38347 | 67.12 |
| 132 | Hylidae | Lophyohylinae | *Osteocephalus* | *Osteocephalus heyeri* | INPA | 38179 | 49.38 |
| 133 | Hylidae | Lophyohylinae | *Osteocephalus* | *Osteocephalus leprieurii* | INPA | 36729 | 58.61 |
| 134 | Hylidae | Lophyohylinae | *Osteocephalus* | *Osteocephalus oophagus* | INPA | 38513 | 98.97 |
| 135 | Hylidae | Lophyohylinae | *Osteocephalus* | *Osteocephalus planiceps* | INPA | 38330 | 66.98 |
| 136 | Hylidae | Lophyohylinae | *Osteocephalus* | *Osteocephalus taurinus* | INPA | 25377 | 103.16 |
| 137 | Phyllomedusidae |  | *Phyllomedusa* | *Phyllomedusa bicolor* | NHMUK | 19202111 | 103.25 |
| 138 | Phyllomedusidae |  | *Phyllomedusa* | *Phyllomedusa bicolor* | NHMUK | 193772945 | 76.12 |
| 139 | Phyllomedusidae |  | *Phyllomedusa* | *Phyllomedusa bicolor* | NHMUK | 18721016111 | 114.28 |
| 140 | Phyllomedusidae |  | *Phyllomedusa* | *Phyllomedusa boliviana* | NHMUK | 1968501 | 60.90 |
| 141 | Phyllomedusidae |  | *Phyllomedusa* | *Phyllomedusa coelestis* | NHMUK | 18748495 | 62 |
| 142 | Phyllomedusidae |  | *Phyllomedusa* | *Phyllomedusa distincta* | NHMUK | 1895367 | 43.88 |
| 143 | Phyllomedusidae |  | *Phyllomedusa* | *Phyllomedusa distincta* | NHMUK | 192361100 | 55.98 |
| 144 | Phyllomedusidae |  | *Phyllomedusa* | *Phyllomedusa distincta* | NHMUK | 192361101 | 49 |
| 145 | Phyllomedusidae |  | *Phyllomedusa* | *Phyllomedusa distincta* | NHMUK | 1925115108 | 50.66 |
| 146 | Phyllomedusidae |  | *Phyllomedusa* | *Phyllomedusa iheringii* | NHMUK | 19702035 | 52.30 |
| 147 | Phyllomedusidae |  | *Phyllomedusa* | *Phyllomedusa iheringii* | NHMUK | 188842322 | 59.46 |
| 148 | Phyllomedusidae |  | *Phyllomedusa* | *Phyllomedusa iheringii* | NHMUK | 188842323 | 51.05 |
| 149 | Phyllomedusidae |  | *Phyllomedusa* | *Phyllomedusa sauvagii* | NHMUK | 18987724 | 68.34 |
| 150 | Phyllomedusidae |  | *Phyllomedusa* | *Phyllomedusa sauvagii* | NHMUK | 19281812 | 76.44 |
| 151 | Phyllomedusidae |  | *Phyllomedusa* | *Phyllomedusa sauvagii* | NHMUK | 190852928 | 64.72 |
| 152 | Phyllomedusidae |  | *Phyllomedusa* | *Phyllomedusa tarsius* | NHMUK | 190251525 | 86.12 |
| 153 | Phyllomedusidae |  | *Phyllomedusa* | *Phyllomedusa tarsius* | NHMUK | 190410291951991 | 68.23 |
| 154 | Phyllomedusidae |  | *Phyllomedusa* | *Phyllomedusa trinitatis* | NHMUK | 19711629 | 71.50 |
| 155 | Phyllomedusidae |  | *Pithecopus* | *Pithecopus ayeaye* | NHMUK | 1988140 | 41.17 |
| 156 | Phyllomedusidae |  | *Pithecopus* | *Pithecopus palliatus* | INPA | 30201 | 39.68 |
| 157 | Hylidae | Acridinae | *Pseudacris* | *Pseudacris regilla* | NHMUK | 504228 | 32.50 |
| 158 | Hylidae | Acridinae | *Pseudacris* | *Pseudacris regilla* | NHMUK | 85115911 | 35.85 |
| 159 | Hylidae | Scinaxinae | *Scinax* | *Scinax boesemani* | INPA | 26621 | 29.72 |
| 160 | Hylidae | Scinaxinae | *Scinax* | *Scinax boesemani* | INPA | 26622 | 30 |
| 161 | Hylidae | Scinaxinae | *Scinax* | *Scinax boesemani* | INPA | 26623 | 31.1 |
| 162 | Hylidae | Scinaxinae | *Scinax* | *Scinax funereus* | INPA | 26668 | 26.44 |
| 163 | Hylidae | Scinaxinae | *Scinax* | *Scinax garbei* | INPA | 28700 | 48.40 |
| 164 | Hylidae | Scinaxinae | *Scinax* | *Scinax garbei* | INPA | 28701 | 47.80 |
| 165 | Hylidae | Scinaxinae | *Scinax* | *Scinax garbei* | INPA | 28702 | 50.09 |
| 166 | Hylidae | Scinaxinae | *Scinax* | *Scinax nebulosus* | INPA | 34641 | 28.33 |
| 167 | Hylidae | Scinaxinae | *Scinax* | *Scinax nebulosus* | INPA | 34650 | 30.66 |
| 168 | Hylidae | Scinaxinae | *Scinax* | *Scinax nebulosus* | INPA | 34653 | 28.54 |
| 169 | Hylidae | Scinaxinae | *Scinax* | *Scinax proboscideus* | INPA | 10304 | 46.86 |
| 170 | Hylidae | Hylinae | *Smilisca* | *Smilisca baudinii* | NHMUK | 1955179 | 66 |
| 171 | Hylidae | Hylinae | *Smilisca* | *Smilisca baudinii* | NHMUK | 19242182 | 52 |
| 172 | Hylidae | Hylinae | *Smilisca* | *Smilisca baudinii* | NHMUK | 19732376 | 55 |
| 173 | Hylidae | Hylinae | *Smilisca* | *Smilisca baudinii* | NHMUK | 19851090 | 60.87 |
| 174 | Hylidae | Hylinae | *Smilisca* | *Smilisca baudinii* | NHMUK | 19851091 | 57.82 |
| 175 | Hylidae | Hylinae | *Smilisca* | *Smilisca baudinii* | NHMUK | 19851092 | 62 |
| 176 | Hylidae | Hylinae | *Smilisca* | *Smilisca baudinii* | NHMUK | 19851093 | 50 |
| 177 | Hylidae | Hylinae | *Smilisca* | *Smilisca baudinii* | NHMUK | 18864154554 | 64.56 |
| 178 | Hylidae | Hylinae | *Smilisca* | *Smilisca baudinii* | NHMUK | 92103181 | 60.77 |
| 179 | Hylidae | Hylinae | *Smilisca* | *Smilisca phaeota* | NHMUK | 1987810 | 52.32 |
| 180 | Hylidae | Hylinae | *Smilisca* | *Smilisca phaeota* | NHMUK | 9542922 | 63.82 |
| 181 | Hylidae | Hylinae | *Smilisca* | *Smilisca phaeota* | NHMUK | 1898313435 | 69.85 |
| 182 | Hylidae | Hylinae | *Smilisca* | *Smilisca phaeota* | NHMUK | 1915102169 | 66.28 |
| 183 | Hylidae | Hylinae | *Smilisca* | *Smilisca phaeota* | NHMUK | 18947265960 | 56.12 |
| 184 | Hylidae | Hylinae | *Smilisca* | *Smilisca phaeota* | NHMUK | 1923101218191 | 60.21 |
| 185 | Hylidae | Hylinae | *Smilisca* | *Smilisca phaeota* | NHMUK | 1913111211812589 | 65.94 |
| 186 | Hylidae | Hylinae | *Smilisca* | *Smilisca phaeota* | NHMUK | 1913111211812593 | 65.42 |
| 187 | Hylidae | Hylinae | *Smilisca* | *Smilisca phaeota* | NHMUK | 19131112118125981 | 64.12 |
| 188 | Hylidae | Hylinae | *Smilisca* | *Smilisca sordida* | NHMUK | 1979374 | 60.89 |
| 189 | Hylidae | Scinaxinae | *Sphaenorhynchus* | *Sphaenorhynchus dorisae* | INPA | 2980 | 25.84 |
| 190 | Hylidae | Lophyohylinae | *Trachycephalus* | *Trachycephalus coriaceus* | INPA | 24591 | 60.31 |
| 191 | Hylidae | Lophyohylinae | *Trachycephalus* | *Trachycephalus cunauaru* | INPA | 39649 | 68.93 |
| 192 | Hylidae | Lophyohylinae | *Trachycephalus* | *Trachycephalus resinifictrix* | INPA | 3796 | 68.6 |
| 193 | Hylidae | Lophyohylinae | *Trachycephalus* | *Trachycephalus resinifictrix* | INPA | 11893 | 75.44 |
| 194 | Hylidae | Lophyohylinae | *Trachycephalus* | *Trachycephalus typhonius* | INPA | 7113 | 76.08 |
| 195 | Hylidae | Lophyohylinae | *Trachycephalus* | *Trachycephalus typhonius* | INPA | 16862 | 74.42 |
